# *Wolbachia* in the Genus *Bicyclus*: a Forgotten Player

**DOI:** 10.1007/s00248-017-1024-9

**Published:** 2017-07-12

**Authors:** Anne Duplouy, Oskar Brattström

**Affiliations:** 1 Metapopulation Research Centre, Department of Biosciences, The University of Helsinki, PL65 Viikinkaari 1, FI-00014 Helsinki, Finland; 2 Department of Zoology, University of Cambridge, Downing Street, Cambridge, CB2 3EJ UK

**Keywords:** Symbiosis, Butterfly, *Bicyclus anynana*, Model organism

## Abstract

*Bicyclus* butterflies are key species for studies of wing pattern development, phenotypic plasticity, speciation and the genetics of Lepidoptera. One of the key endosymbionts in butterflies, the alpha-Proteobacterium *Wolbachia pipientis*, is affecting many of these biological processes; however, *Bicyclus* butterflies have not been investigated systematically as hosts to *Wolbachia*. In this study, we screen for *Wolbachia* infection in several *Bicyclus* species from natural populations across Africa as well as two laboratory populations. Out of the 24 species tested, 19 were found to be infected, and no double infection was found, but both A- and B-supergroup strains colonise this butterfly group. We also show that many of the *Wolbachia* strains identified in *Bicyclus* butterflies belong to the ST19 clonal complex. We discuss the importance of our results in regard to routinely screening for *Wolbachia* when using *Bicyclus* butterflies as the study organism of research in eco-evolutionary biology.

**Electronic supplementary material:**

The online version of this article (doi:10.1007/s00248-017-1024-9) contains supplementary material, which is available to authorized users.

## Introduction

Current estimates suggest that up to 70% of all insect species in the world may live in intimate relation with intracellular micro-organisms [1, 2]. The outcome of such symbiotic associations, or endosymbiosis, ranges from mutualistic and beneficial to both the host and the microbe, to parasitic and detrimental to the host [3]. The bacterial species *Wolbachia pipientis* Hertig, 1936 [4], is one of the most common and best-studied endosymbionts found in insects. This maternally transmitted alpha-Proteobacterium selfishly promotes its own fitness by manipulating several aspects of its host’s biology [5]. The many potential distortions of the host’s fitness include the manipulation of its reproductive system [5] and of various life-history traits, such as fecundity [6], dispersal [7] and resistance to stresses caused by pathogens or the environment [8]. In *Drosophila simulans* for example, the *Wolbachia* strain *w*Ri causes karyogamy failure and the arrest of early embryonic development of the offspring, when *Wolbachia-*free female flies are mated with *Wolbachia-*infected males [9]. However, at the same time, this bacterial strain protects the infected flies from viral infections [10]. By promoting its own fitness, through increasing the fitness of the infected host lines compared to uninfected ones, *Wolbachia* has the potential to act as a key player in the ecology and evolution of its hosts.

Butterflies and moths have long fascinated and attracted the attention of entomologists, both professionals and amateurs. They are generally easily identified compared to many other insect groups, thus facilitating the documentation of their habits and behaviors in natural environments. Furthermore, Lepidoptera often have relatively short life-cycles and high fecundity, and many species can be reared continuously in laboratory environments, thus making their use for large-scale experiments feasible. Finally, with recent development of molecular techniques, the study of insect genetics and genomics is no longer restricted to the *Drosophila* scientific community. The full genomes of 36 Lepidoptera are now publicly available (see for example [11–15]), and more are in preparation or partially sequenced (lepbase.org [16]). The availability of these new tools combined with our extended knowledge of gene to life-history traits, gained from both laboratory and field-based studies, makes many Lepidoptera species suitable model organisms for ecology and evolutionary studies.

There are over 100 species in the butterfly genus *Bicyclus* (Nymphalidae) [17]. Many species have distinct seasonal morphs mediated through environmentally induced plasticity. These seasonal phenotypes are characterised by variations in wing patterns [18, 19], pheromone compounds [20] and various life-history traits [21, 22]. Oliver et al. [23] have, for example, characterised different expression levels for several genes involved in the regulation of the eyespot wing patterns between *B. anynana* specimens developing in different environmental conditions, while Macias-Munoz et al. [24] have shown that sexes and seasonal morphs differentially express several other genes involved in insect vision. Combined together, these results suggest that the environment may play an important role in both mate recognition and choice in *Bicyclus.* The imminent completion of the *B. anynana* full genome sequencing project (lepbase.org) will certainly lead to an increased number of studies further investigating the consequences of seasonality in the evolutionary history of this and other *Bicyclus* species.

Despite the current wealth of eco-evolutionary studies on *Bicyclus* butterflies, and the fact that *Wolbachia* is a symbiont of many butterfly species worldwide [25], we are unaware of a study identifying the bacterium as an endosymbiont of *B. anynana*, or of any other *Bicyclus* species. This knowledge gap currently clearly contrasts with our understanding of peculiar host-symbiont interactions in several related Nymphalidae butterflies. *Wolbachia* are for example present in various species of *Heteropsis* [26], a genus closely related to *Bicyclus* [27], and have also been associated with *Coenonympha tullia* [28], *Maniola jurtina* [29], *Caligo telamonius* [30] and many other butterfly species [25]. Although further studies are needed to fully characterise the effect of *Wolbachia* in their butterfly hosts, the currently available studies on symbiotic interaction in a few butterfly species already suggest that the effects from such infections can have profound consequences for the eco-evolutionary biology of their butterfly hosts. The *Wolbachia* strains infecting the butterflies *Hypolimnas bolina* and *Acraea encedon*, for example, kill the male progeny of infected female butterflies [31, 32], thus potentially limiting inbreeding and sibling resource competition in these species.

In this paper, we report on the results from a screening for presence of *Wolbachia* infections, and the diversity of the *Wolbachia* strains, in samples from natural populations of several *Bicyclus* species from across sub-Saharan Africa. The characterisation of these *Wolbachia* strains is based on the standardised use of the five multilocus sequence typing markers (MLSTs [33]), and the genetic marker *wsp* [34]. For the first time, we show that *Wolbachia* infections are common in *Bicyclus* species, and that the strain diversity within this host genus is relatively high. We discuss the potential implications of these results for future studies using *Bicyclus* butterflies as focal organisms.

## Material and Method

### Material

The *Bicyclus* genus represents a highly diverse genus of over 100 African butterfly species [17]. The majority of the species inhabits the rainforest zone, while others are found in the savannah regions [35]. The many strikingly morphologically similar species in the genus are often only distinguishable through the comparison of highly divergent wing scales called androconia, specialised structures at least partly linked to pheromone production and release [36].

In total, 200 specimens from 24 species across the genus *Bicyclus* were used in this study [17] (Table 1). To avoid phylogenetic bias, we included samples from all except one (*ena*-group) of the 16 species groups currently recognised in the genus [17]. Nineteen of the sampled species generally inhabit forest environments, but some will also tolerate dry forest environments, while the remaining five are generally found in savannah habitats. Most species were collected from wild populations between 2008 and 2012; however, *B. anynana* and *B. safitza* samples were collected in 2016 from matrilines that had been maintained for several generations in the laboratory. The samples of *B. anynana* originated from a stock of around 80 females collected in Malawi in 1988 and currently reared by many labs across the world. The samples of *B. safitza* come from a more recent stock established from ten females collected in Uganda in 2013. The species identities of our specimens were previously identified for the purpose of a phylogenetic study by Aduse-Poku et al. [17]. No species of *Bicyclus* are currently classified as threatened species, and all field-collected samples were collected with permission from governmental organisations in their countries of origin. For each species except one (see below), four to ten individuals were tested for infection by *Wolbachia*. Since we focused on the screening of samples from a range of locations (up to five when possible) for each species, we potentially maximised the chance of detecting infections as well as strain diversity per species, but not per population*.* Finally, since we had no prior knowledge with regards to the induction of male killing by endosymbionts in *Bicyclus*, we privileged female samples, but included males to make up the numbers when we did not have enough females available. Full details of all samples are available in Supplementary material Table S1.

**Table 1:** Country of origin, *Wolbachia* infection penetrance and strain type in the 24 *Bicyclus* species investigated in this study. "UnSt" stands for Uncharacterised Strain

Host species	Country	Infection rate (infected/uninfected)	Strain ID (strain type ST no.)
*B. anisops*	Nigeria	90% (9/1)	*w*Bani_B
*B. anynana anynana*	Mala*w*i	0%	–
*B. auricruda fulgida*	Nigeria	60% (6/4)	*w*Baur2_A (ST19) + *w*Baur_A + 1-UnSt
*B. collinsi*	Uganda	86% (6/1)	*w*Bcol_A (ST19)
*B. dentata*	Uganda	87.5% (7/1)	*w*Bden_B
*B. ephorus*	Liberia Nigeria	100% (5/0) 60% (3/2)	*w*Beph_B *w*Beph_B
*B. evadne*	Liberia Nigeria	86% (6/1) 100% (1/0)	*w*Beva_A + *w*Beva_B *w*Beva_B
*B. funebris*	Nigeria	70% (7/3)	*w*Bfun_B + 1-UnSt
*B. ignobilis*	Nigeria	100%	*w*Bign_A (ST19)
*B. italus*	Nigeria	70% (7/3)	*w*Bita_B
*B. jacksoni*	Liberia	100% (4/0)	*w*Bjac_B (ST187)
*B. mandanes*	Nigeria	100% (1/0)	*w*Bman_A (ST19)
*B. nobilis*	Nigeria	100% (8/2)	*w*Bnob_B
*B. pavonis*	Nigeria	28.5% (2/5)	*w*Bpav_B (ST423) + 1-UnST
*B. procora*	Ghana	0% (0/5)	–
*B. safitza safitza*	Uganda	0% (0/10)	–
*B. sanaos*	Nigeria	100% (10/0)	2-UnSt
*B. sangmelinae*	Ghana Liberia	0% (0/4) 67% (4/2)	– *w*Beph_B
*B. smithi smithi*	Nigeria	0% (0/10)	–
*B. sylvicolus*	Nigeria	10% (1/9)	*w*Bsyl_B
*B. taenias*	Ghana Liberia Nigeria	100% (1/0) 80% (4/1) 100% (4/0)	*w*Btae_B *w*Btae_B *w*Btae_B
*B. vulgaris*	Ghana Liberia Nigeria	0% (0/3) 0% (0/1) 50% (3/3)	– – *w*Bvul_A (ST19) + 1-UnSt
*B. xeneas*	Ghana Liberia Nigeria	100% (1/0) 100% (2/0) 86% (6/1)	1-UnSt *w*Bxen_A + *w*Bxen2_A *w*Bxen_A + *w*Bxen2_A
*B. xeneas xeneas*	Nigeria	50% (1/1)	*w*Bxen_B
*B. xeneoides*	Nigeria	0% (0/5)	–

Note that during the sequencing phase of this project, one of the species included in our original dataset, *B. mandanes*, was taxonomically revised and split in two distinct species [17]. This affected our data set that originally included ten samples of *B. mandanes*, with nine of these being moved to the newly discovered species *B. collinsi* and only one single specimen remained as *B. mandanes*.

### Screening for *Wolbachia* infections

We separated the wings from the bodies of the *Bicyclus* specimens immediately after collection in the field, and the tissues were individually preserved in Eppendorf tubes filled with 99% ethanol, kept in a fridge until further dissection. For each butterfly, we extracted the DNA from thoracic tissues using Qiagen DNeasy Blood & Tissue Extraction Kit, and following the manufacturer’s protocol (Qiagen, USA). We tested the quality of all DNA extracts by PCR amplification of the *COI* gene (primer pair LCO/HCO, [37]). We tested for *Wolbachia* infection by PCR using five MLST markers (multilocus sequence typing genes: *coxA*, *fbpA*, *ftsZ*, *gatB* and *hcpA*, using respective primers from [33], and the *wsp* gene (primer pairs 81F/691R [34]). All amplified sequences were deposited into GenBank (#KY658538-664).

### Genotyping the *Wolbachia* strains

Amplicons from each MLST and the *wsp* genes were sequenced on an automated ABI 3730 DNA Analyzer (Applied Biosystems™, USA). Both reverse and forward strand were sequenced for each sample. Sequences were manually curated using Geneious R6 (http://www.geneious.com [38]) for consistency between reverse and forward sequences of each sample. The lack of double nucleotide picks along the sequences supported single *Wolbachia* infection in our samples. All sequences were compared to the PubMLST database (http://pubmlst.org/wolbachia [33]) using BLASTn. New allelic profiles were manually curated, characterised and added to the PubMLST database. Note that we used *Wolbachia* primers previously designed by Baldo et al. [33] in non-optimised conditions, and failed to amplify all MLST loci for several strains, thus providing new evidence that the currently available tools are not optimum for a universal genotyping of the *Wolbachia* strains in Lepidoptera. For the purpose of this study, we assigned a full strain name when three or more of the MLST loci were fully sequenced, and a potential strain name when only two MLST loci were sequenced, but did not assign any strain name if only one MLST locus was sequenced, whether or not the *wsp* gene was sequenced.

### Phylogeny

**Fig. 1:**
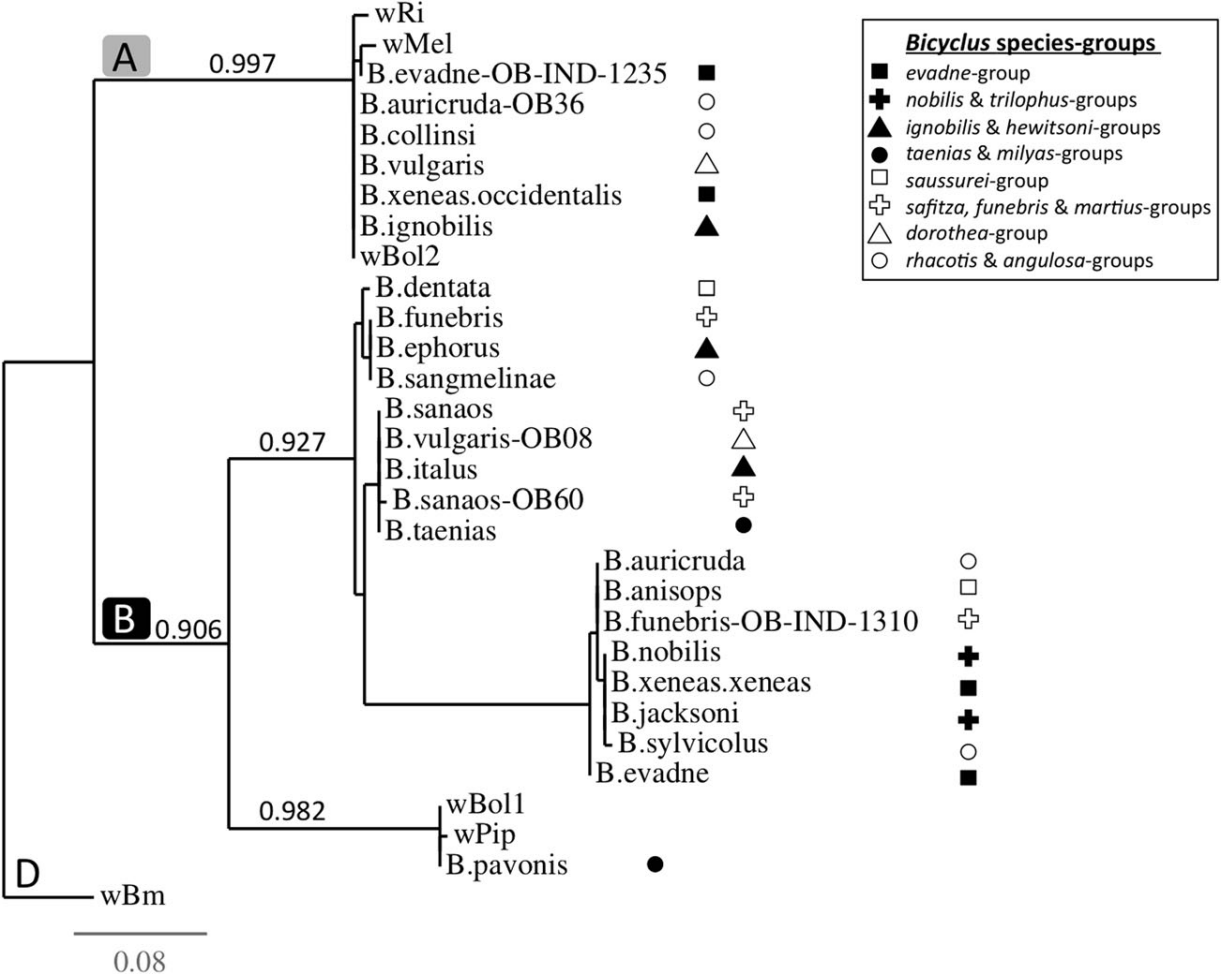
Rooted phylogram based on the different allelic profiles of the *coxA* gene amplified from *Bicyclus* butterflies and six reference strains (*w*Mel, *w*Ri, *w*Bol2, *w*Pip, *w*Bol1 and *w*Bm), with PhyML aLRT-based branch support values. *A*, *B* and *D* refer to three *Wolbachia* supergroups. Branches are named after the host species names, and sample ID when necessary. The *w*Bm strain from the parasitic nematode *Brugia malayi* was used as the outgroup. The *Bicyclus* hosts have been labelled according to their respective species groups (as defined in [17]) and show no clear pattern of congruence with the *Wolbachia* phylogeny

The *Wolbachia* phylogenetic trees were built using the online tree-building program *Phylogeny*.*fr* in the *One-click* mode with default settings [39]. In brief, the *One-Click* method builds a maximum likelihood tree using PhyML [40] with sequence alignment using MUSCLE [41, 42]. We used the coxA marker to build our main *Wolbachia* phylogenetic tree (Fig. 1), as this locus was amplified and sequenced for the largest number of *Bicyclus* samples and species. Similarly, we used the MLST sequences from each of the 13 strains that were fully characterised, to build the phylogenetic tree of these *Wolbachia* strain (Fig. S1). The respective MLST sequences from six additional *Wolbachia* strains (*w*Bol2, *w*Mel, *w*Ri, *w*Bol1, *w*Pip and *w*Bm; GenBank nos. AM999887, CP001391, AE017321, AE017196, EF025179–183, EF078895, AB474245–249, AB094382), previously characterised as belonging to the A-, B- or D-supergroups, were added to the trees. We rooted the trees using *w*Bm as the outgroup.

### Statistics

Due to our restricted sample size in certain categories, we decided to independently test for sex and habitat effects on the *Wolbachia* infection status of these butterflies using chi-square tests.

## Results

### Prevalence and Penetrance

In total, 19 (79.2%) out of the 24 species tested were found infected by *Wolbachia* (*N*_inf_ = 113 butterflies) (Table 1). Infection rates ranged from 10 to 100% depending on the species and the country of origin of the samples tested (Table 1). The infection rate did not differ between males (56.1%, *N*_inf./tot._ = 37/66) and females (56.7%, *N*_inf./tot_ = 76/134) (*χ* ^2^ = 0.008, df = 1, *P* = 0.9299). Comparing samples from savannah- and forest-related habitats, there was a significantly higher infection ratio in samples from forest (64.7%, *N*_inf./tot._ = 99/153) compared to savannah (29.8%, *N*_inf./tot_ = 14/47) (*χ* ^2^ = 17.838, df = 1, *P* = 0.0001). It should be noted that all samples from two of the savannah-adapted species (*N* = 20) originated from lab stocks were free of infections. Given their rearing conditions, they might not demonstrate a natural infection rate (see “Discussion” section). When these laboratory-reared samples are removed from our analysis, the difference between habitats is no longer significant (*χ* ^2^ = 1.623, df = 1, *P* = 0.2027). The dataset however becomes highly biased with almost all samples (153 out of 180) from forest species.

### Phylogenetic Congruence

Amplification of the *wsp* and of all five MLST loci was not always successful, and therefore, a thorough phylogenetic analysis based on the concatenated sequences of all markers could not be completed. The results of our amplification efforts can be found in Table S1. The *coxA* locus successfully amplified for most of our *Wolbachia*-infected butterflies but 13 specimens. Visual comparative analysis of symbiotic strains versus host phylogenetic clustering shows no congruence between the phylogenies of the butterfly hosts and of their *Wolbachia* symbionts, with phylogenetically close *Wolbachia* strains being found in phylogenetically distant *Bicyclus* host species. This is true when we used either the *coxA* sequence only (Fig. S1) or the concatenated *wsp* and MLST sequences (Fig. S1) for building the *Wolbachia* phylogenies.

### *Wolbachia* strain diversity

All strains except one were exclusively found in a single *Bicyclus* species each, the exception being strain ST19 that was found in five species (*B. auricruda*, *B. collinsi*, *B. ignobilis*, *B. mandanes* and *B. vulgaris*). Additionally, the within-host species symbiotic strain diversity was low, with generally a single strain detected per species. However, in six species more than one strain was detected. The highest diversity was found in *B. auricruda* and *B. xeneas*, where three separate strains were at least partially characterised in each species. We did not detect any double infection in any of our samples.

Notably, the three strains (one uncharacterised strain, *w*Baur_A and *w*Baur2_A/ST19) infecting *B. auricruda fulgida* were found at a single sample site (Ologbo Forest, Nigeria). In contrast, the three strains found in *B. xeneas* show a more geographical separation with strain *w*Bxen_A found in Liberia, Ghana and Western Nigeria (Ologbo and Omo forests), strain *w*Bxen2_A found in Liberia only and strain *w*Bxen_B from Rhoko in Eastern Nigeria. Two strains were also found in the species *B. evadne* (*w*Beva_B and *w*Beva_A), and two strains were partially characterised in *B. funebris* (wBfun_B and one uncharacterised strain), *B. pavonis* (one uncharacterised strain and *w*Bpav_B/ST423) and *B. vulgaris* (one uncharacterised strain and *w*Bvul_A/ST19), again with different strains often being detected at the same sample sites.

Most of our *B. xeneas* specimens carry *Wolbachia* strains that can be grouped to the same clonal complex (STC-19), because these strains share at least three identical loci with ST19. The strains from the STC-19 were found in specimens collected from sites ranging from across the African rainforest belt (from Sapo, Liberia, through Ghana and Nigeria to Kibale in Uganda). This wide-ranging occurrence supports the findings of Ahmed et al. [25], which suggested that the strains from the STC-19 are capable of inter-familial, inter-superfamilial and inter-ordinal horizontal transmission. The strain ST19 was previously characterised from several other species of Lepidoptera, including *Ephestia kuehniella*, *Aricia artaxerxes* and *Ornipholidotos peucetia*, as well as various species of Hymenoptera and Coleoptera (pubmlst.org). Similarly, the strain ST187 found in *B. jacksoni* and the strain ST423 found in *B. pavonis* were both previously characterised from the endoparasitoid wasp *Diaphorencyrtus aligarhensis*, and an unspecified host species, respectively (pubmlst.org). All other strains, but not all allelic profiles of each marker, were new to the PubMLST database.

As often observed in Lepidoptera, *Bicyclus* butterflies serve as hosts to both A-supergroup *Wolbachia* strains (*N*_A_ = 30, from eight host species) and/or B-supergroup *Wolbachia* strains (*N*_B_ = 82, from 16 host species). Recombination is rampant in *Wolbachia* [43], and our dataset suggests past exchanges of loci within the A- and B-supergroups (Table S1). Both strains *w*Beva_A and *w*Bing_A share the same allelic profile at the ftsZ locus, while all other loci are divergent; similarly, *w*Bpav_B and *w*Btae_B only share an identical fbpA allelic profile (Fig. S2). In contrast, we did not find any recombining strains, with an admixture of loci from the A- and B-supergroups. Note that for the *gatB* locus sequenced from one sample of the *B. nobilis* species, the resulting sequence grouped in the A-supergroup, while all other locus groups are in the B-supergroup; however, our consecutive attempts at re-sequencing this particular locus failed, therefore we do not consider this as a reliable result. In contrast, we found both A- and B-supergroup strains in separate specimens from three species (*B. auricruda*, *B. evadne* and *B. xeneas*). Notably, both supergroups were found in similar proportions in males (*N*_a/b_ = 6/31) and females (*N*_a/b_ = 24/51) (*χ* ^2^ = 3.1475, df = 1, *P* = 0.0760).

## Discussion

*Wolbachia* is highly prevalent in the butterfly genus *Bicyclus*, with penetrance of the infection in each species and population often reaching 50% or higher. We detected the presence of the endosymbiotic bacterium *Wolbachia* in 113 butterflies (56.5%) from 19 (79.2%) of the 24 tested species of the genus *Bicyclus*. However, because not all our samples successfully amplified with each marker used, and due to our relatively small sample size for each butterfly species and population, it is possible that we are still under-estimating the true prevalence and diversity of *Wolbachia* in the genus *Bicyclus*. Notably, the absence of *Wolbachia* in the laboratory stocks of *B. anynana* and *B. safitza* could also be the outcome of directed selection due to the artificial long-term rearing environment, which may have for unknown reasons favoured uninfected matrilines. In contrast, the potential occurrence of the horizontal transfer of *Wolbachia* genes to the host genomes [44] may lead to an over-estimation of the prevalence of cytoplasmic *Wolbachia* in these butterflies. However, since nuclear copies of *Wolbachia* genes would evolve in a different way than cytoplasmic copies, we suggest that the same allelic profiles should not be found in highly divergent butterfly hosts, as we currently observe. Although none of the loci tested in this study were amplified from *B. anynana* samples, loci could be identified in the whole genome sequence of this host species by screening for genes that indicate transfer of *Wolbachia* genomic material to the host genome.

The strain diversity within each *Bicyclus* species is generally low, with three or fewer strains described in each host species. Populations of *B. xeneas* were infected by different *Wolbachia* strains, correlating to a subspecific border in this species, with the Western samples (*B. xeneas occidentalis*) carrying *Wolbachia* strains from the A-supergroup ST19-clonal complex, while the Eastern sample (*B. x. xeneas*, Rhoko population) carries a yet unidentified, but clearly different, strain from the B-supergroup. In contrast, the strain diversity within the genus *Bicyclus* appears relatively high, with 20 strains fully or partially described in this study. In contrast to recent studies showing that the strain ST41 might be the core strain or ancestral strain of *Wolbachia* in Lepidoptera [25, 43, 45], our study would suggest that ST19 might play such a role in the *Bicyclus* clade. Only two of the *Wolbachia* strains described in *Bicyclus* share at least one locus with ST41 (coxA:14 and fbpA:4 are found in ST423 from *B. pavonis*, and fbpA:4 is also found in the strain infecting *B. taenias*), while six species carry a strain related to the ST19 clonal complex. These results challenge the idea of worldwide similarity of Lepidopteran *Wolbachia* suggested by Ahmed et al. [25], and Ilinsky and Kosterin [43]; however, further analyses of *Wolbachia* diversity in the entire *Bicyclus* clade are needed.

Only two strains, ST19 and *w*Beph_B, were found to infect either closely related (*B. manandes* and *B. collinsi*) or highly divergent host species (*B. ephorus* and *B. sangmelinae*), respectively*.* The fact that phylogenetically diverse *Bicyclus* species share similar *Wolbachia* strain supports a lack of congruence between the hosts and the bacterial strain phylogenies. The acquisition of *Wolbachia* in many *Bicyclus* butterflies therefore potentially happens through horizontal transfer between host species. The mechanisms driving the horizontal transfer of cytoplasmic *Wolbachia* between host species are yet poorly understood. Horizontal transfer of *Wolbachia* was previously suggested between Diptera species feeding on the same mushroom species [46], or between interacting species within food webs (e.g. host-parasitoid food webs [47]). Many *Bicyclus* species are found in sympatry in similar natural habitats, exhibit similar wing patterns and share relatively similar host plants for feeding and oviposition. Thus, although there are currently no documented records of naturally occurring interspecies hybridisation between *Bicyclus* species, such events may occur more often than previously thought or observed. Interestingly, various strains belonging to the ST19-clonal complex and found in highly divergent *Bicyclus* species were also previously characterised from other butterfly and insect species, including parasitoid wasps. These particular strains may be highly mobile not only between *Bicyclus* species but also at a larger scale between various insect species. A more extensive analysis of the *Wolbachia* infections occurring in the *Bicyclus* butterflies and their associated parasitoids would further inform on the potential origins and transfer routes of these symbionts in the wild populations of the butterfly hosts. Similarly, future studies with larger number of samples, especially with more males and species from the savannah habitat, should more accurately identify whether sex, habitat or the interaction of the two factors may also correlate with the prevalence of *Wolbachia* in these *Bicyclus* species.

To our knowledge, this study is the first evidence of the presence of *Wolbachia* in *Bicyclus* butterflies. Our results provide complementary information to studies that have detected *Wolbachia* in various other butterfly species [25], as well as a novel angle of investigation to eco-evolutionary research conducted using *Bicyclus* butterflies as model organisms. *Wolbachia* is particularly well-known for its ability to alter its host reproductive system, either through cytoplasmic incompatibility (CI), feminisation, or male killing (MK). The tropical butterfly *H. bolina* is infected by a *Wolbachia* strain inducing MK, a type of symbiont-induced early death of the male progeny of infected females. In the beginning of the century, some populations of *H. bolina* exhibited a sex ratio highly biased in favour of females [48]. The pressures from male rareness in these populations were such that the host species evolved to suppress the symbiont-induced MK phenotype [49, 50], and to re-establish a balanced sex/ratio [51]. Similarly, the African butterfly *A. encedon* carries another MK *Wolbachia* strain. Instead of evolving a suppressor gene like in *H. bolina*, this species shows altered mating behaviors [32]. In uninfected populations of *A. encedon*, the females are the choosing sex visiting hill-topping swarms of males, while in *Wolbachia-*infected populations, a sex role reversal is observed, and males chose between hill-topping females [32]. In most *Bicyclus* species tested, we showed that both male and female specimens could be infected by *Wolbachia*, either suggesting that the symbiotic strains do not induce MK, or that these butterfly species have evolved to repress the MK phenotype.

Furthermore, *Wolbachia* can also affect various life-history traits of its host, including viral protection in flies and mosquitoes [10, 52], mate recognition in *Drosophila* flies [53], sex determination in *Pieridae* butterflies [54] and terrestrial isopods [55, 56], ovarian development in Hymenoptera [57–59] and Nematodes [60] and many more traits in various other insect species. Although research using *Bicyclus* butterflies first focused on the evolution of developmental plasticity, these butterflies are now commonly considered as model organisms for studies in various eco-evolutionary processes [61]. *Bicyclus anynana*, for example, is used to investigate the underlying mechanisms of phenotypic variation and seasonal polyphenism (e.g. using diversity in wing patterns, morphology and other life-history traits [62]), of speciation (e.g. using phylogenetic analyses of almost 100 species [17], or through host plant preference and mate choice experiments [63]), of inbreeding depression [64] and of aging [21]. Whether the *Wolbachia* strains described in this study can alter any fitness trait of their respective host remains unknown. However, with the increasing number of studies now suggesting an important role of symbionts in many animal speciation events [65, 66], either through pre- or post-mating isolation mechanisms, it is not farfetched to propose *Wolbachia* as a potential serious key player in speciation processes within the *Bicyclus* clade. Developing an efficient detection method for *Wolbachia* in the *Bicyclus* clade will allow the fast and full characterisation of all divergent strains associated to these butterflies, which we were currently unable to achieve, and the detection of low titre infections that are often only detected through more time-consuming methods than PCR, such as qPCR or microscopy techniques. Only then might routine *Wolbachia* screenings in *Bicyclus* butterflies become universally feasible, and will enable us to comprehensively highlight the roles played by these potentially hidden factors in eco-evolutionary studies.

## Electronic supplementary material

Fig S1 **Rooted phylogram based on the concatenated sequences of the five MLST (** ***coxA, fbpA, ftsZ, gatB*** **and** ***hcpA*** **) markers from 13** ***Wolbachia*** **strains fully characterised from** ***Bicyclus*** **butterflies, and six reference strains** (*w*Mel, *w*Ri, *w*Bol2, *w*Pip, *w*Bol1, and *w*Bm), PhyML aLRT-based branch support values**.** A, B and D refer to three *Wolbachia* supergroups. Branches are named after the host species names, and sample ID when necessary. The *w*Bm strain from the parasitic nematode *Brugia malayi* was used as outgroup. (GIF 78.3 kb).

High resolution image (TIFF 6376 kb).

Fig S2 **Rooted phylograms based on the different allelic profiles of the (a)** ***wsp,*** **(b)** ***fbpA,*** **(c)** ***ftsZ,*** **(d)** ***gatB*** **and (e)** ***hcpA Wolbachia*** **marker characterised from** ***Bicyclus*** **butterflies, and six reference strains** (*w*Mel, *w*Ri, *w*Bol2, *w*Pip, *w*Bol1, and *w*Bm)**,** PhyML aLRT-based branch support values**.** A, B and D refer to three *Wolbachia* supergroups. The *w*Bm strain from the parasitic nematode *Brugia malayi* was used as outgroup. (GIF 86 kb).

High resolution image (TIFF 3908 kb).

Table S1 **Origin, sex,** ***Wolbachia-*** **infection status and the different allelic profiles for** ***wsp, coxA, fbpA, ftsZ, gatB*** **and** ***hcpA Wolbachia*** **genes from all specimens from the 24 species of** ***Bicyclus*** **butterflies investigated in this study.** (−) no PCR amplification, (+) PCR amplification but failed sequencing. Allelic profiles in brackets are the closest matches to our sequences in the pubmlst.org database. (XLS 87 kb).
