# Supplementary figures and images for: Wolbachia in the Genus Bicyclus: a Forgotten Player
Source: Microb Ecol. 2017 Jul 12;75(1):255–63. doi: 10.1007/s00248-017-1024-9 (PMC5742604; doi:10.1007/s00248-017-1024-9)

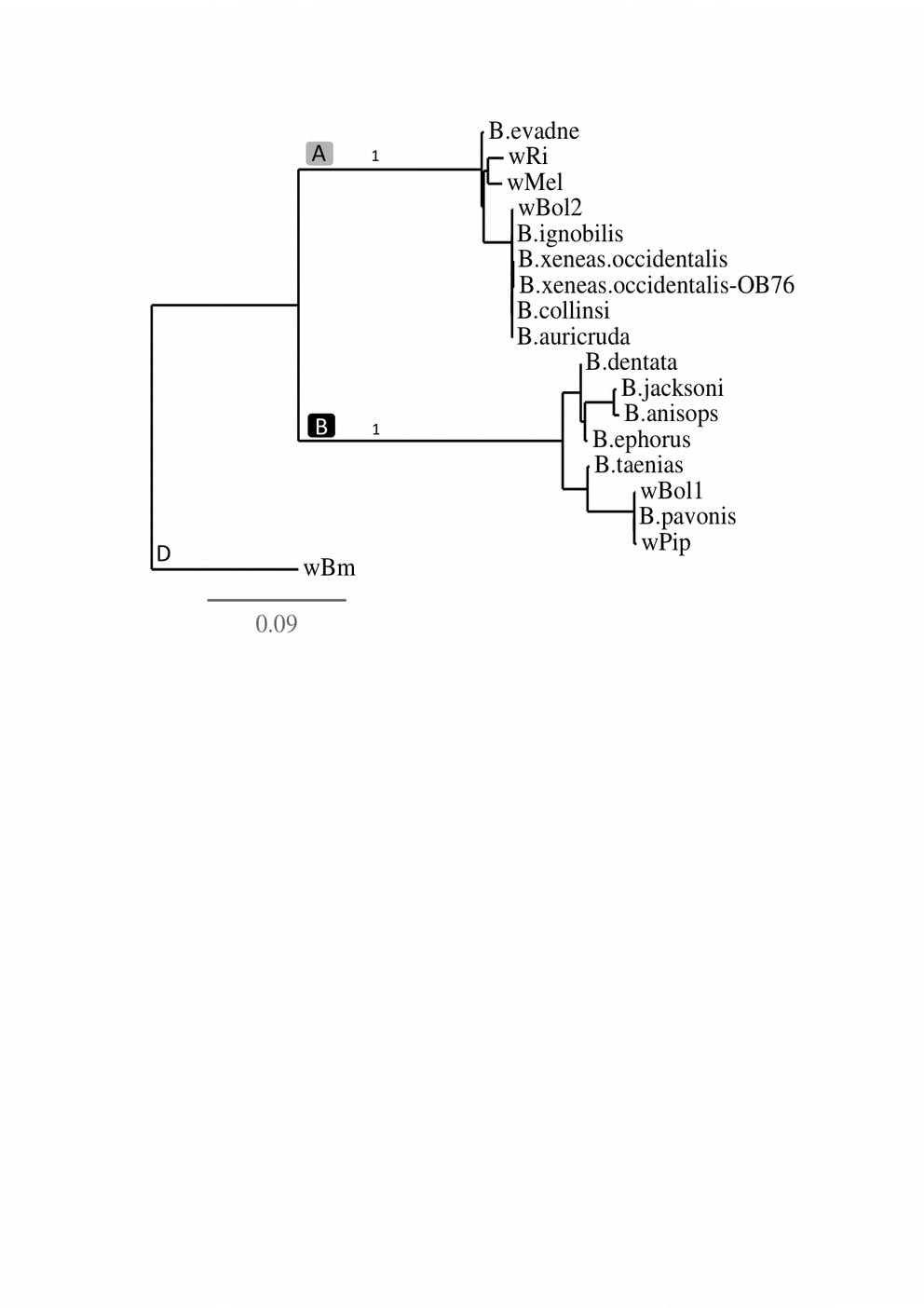

Supplement: Supplementary file 1 — Rooted phylogram based on the concatenated sequences of the five MLST ( coxA, fbpA, ftsZ, gatB and hcpA ) markers from 13 Wolbachia strains fully characterised from Bicyclus butterflies, and six reference strains (wMel, wRi, wBol2, wPip, wBol1, and wBm), PhyML aLRT-based branch support values. A, B and D refer to three Wolbachia supergroups. Branches are named after the host species names, and sample ID when necessary. The wBm strain from the parasitic nematode Brugia malayi was used as outgroup. (GIF 78.3 kb). [file 248_2017_1024_Fig2_ESM.gif]

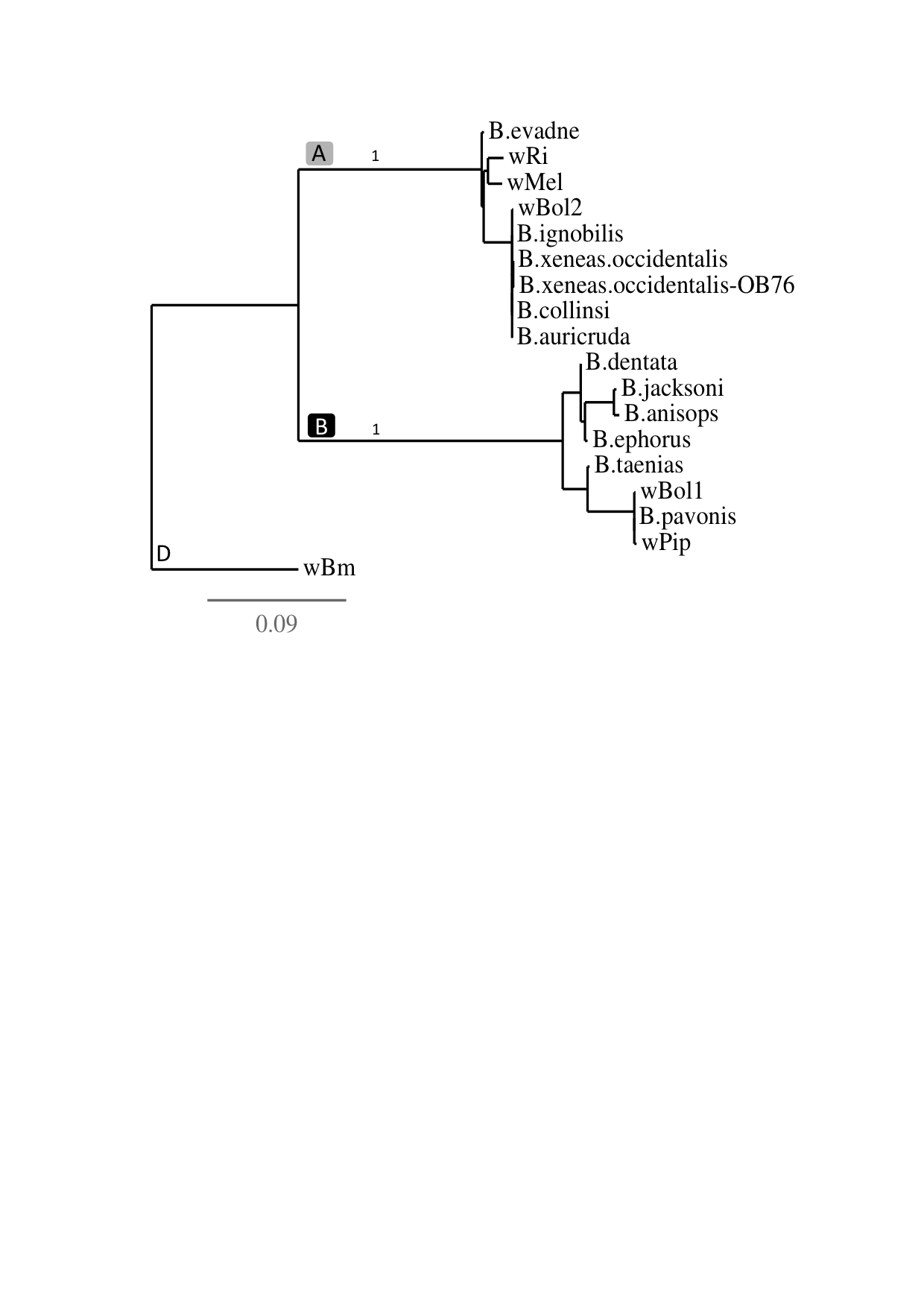

Supplement: Supplementary file 2 — High resolution image (TIFF 6376 kb). [file 248_2017_1024_MOESM1_ESM.tiff]

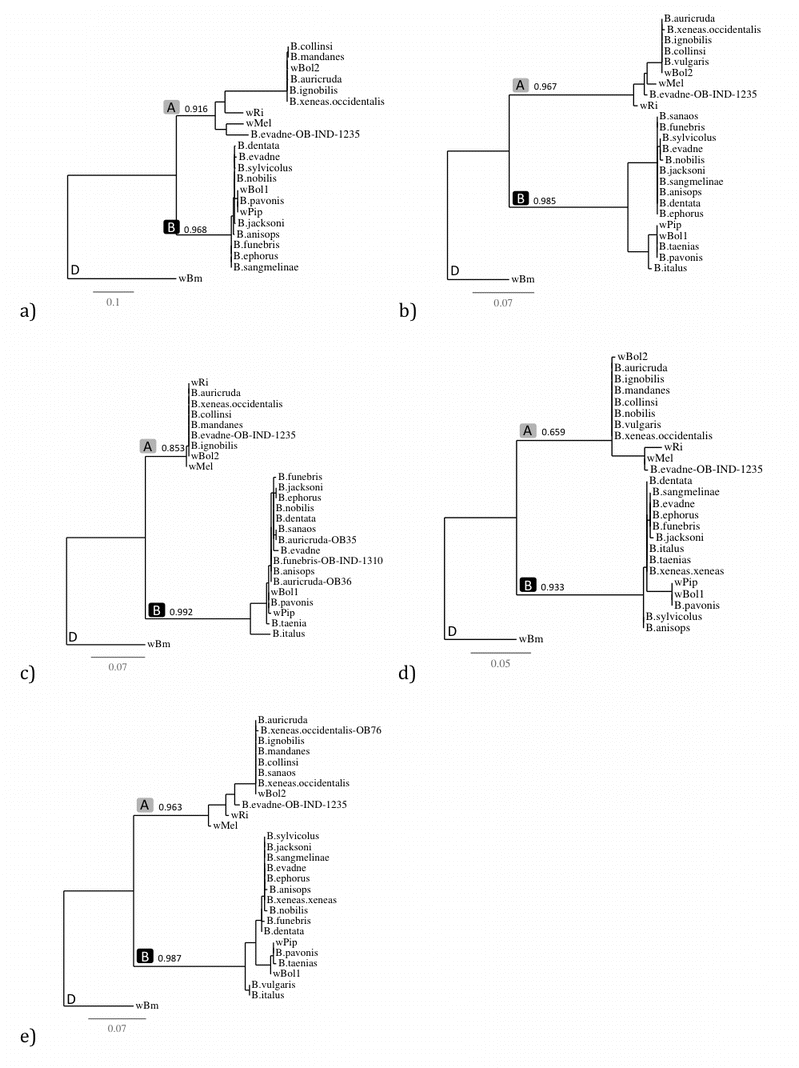

Supplement: Supplementary file 3 — Rooted phylograms based on the different allelic profiles of the (a) wsp, (b) fbpA, (c) ftsZ, (d) gatB and (e) hcpA Wolbachia marker characterised from Bicyclus butterflies, and six reference strains (wMel, wRi, wBol2, wPip, wBol1, and wBm), PhyML aLRT-based branch support values. A, B and D refer to three Wolbachia supergroups. The wBm strain from the parasitic nematode Brugia malayi was used as outgroup. (GIF 86 kb). [file 248_2017_1024_Fig3_ESM.gif]

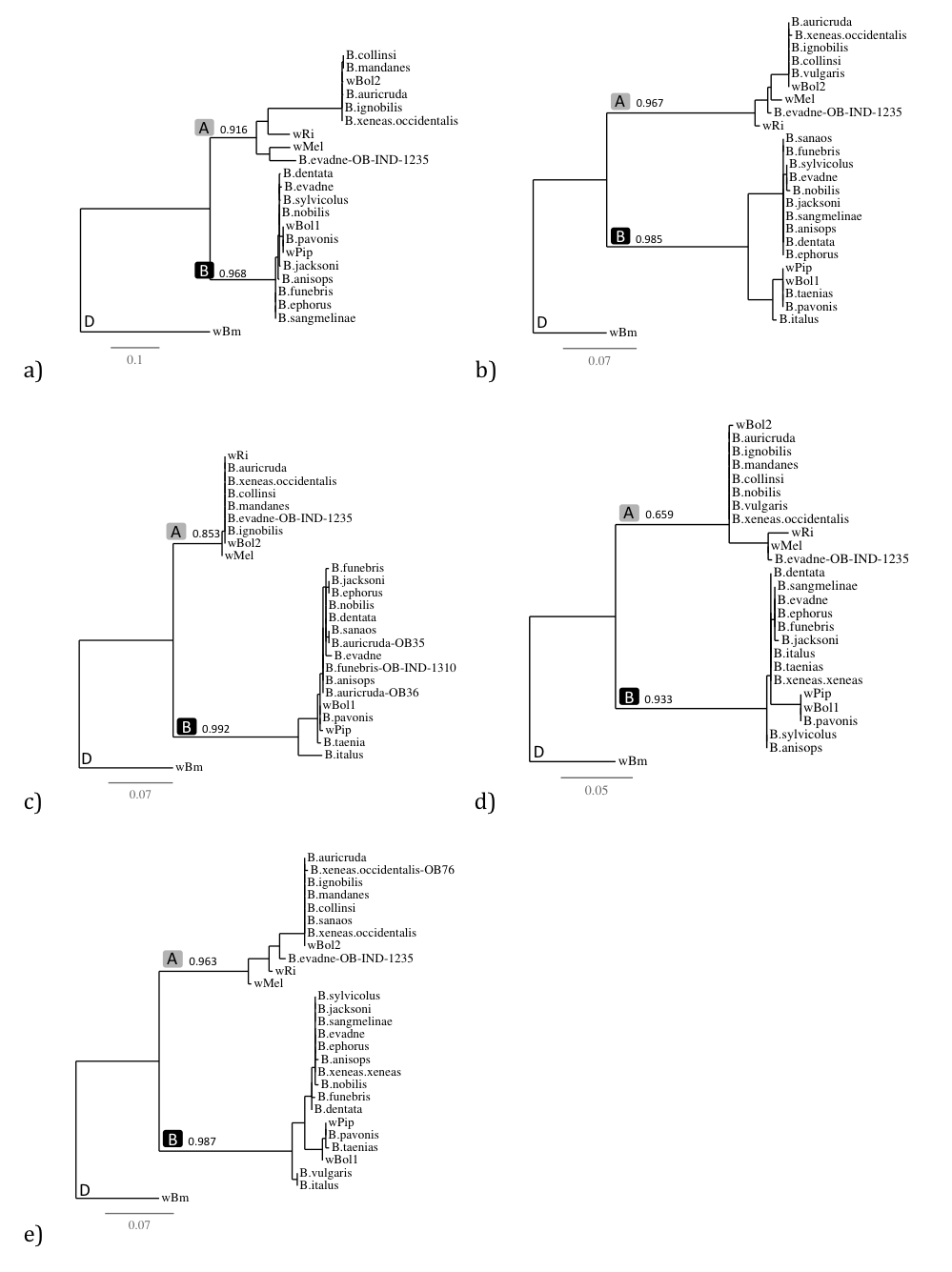

Supplement: Supplementary file 4 — High resolution image (TIFF 3908 kb). [file 248_2017_1024_MOESM2_ESM.tiff]
